# On the Assessment of Strawberries’ Shelf-Life and Quality, Based on Image Analysis, Physicochemical Methods, and Chemometrics

**DOI:** 10.3390/foods13020234

**Published:** 2024-01-11

**Authors:** Georgia Ladika, Irini F. Strati, Thalia Tsiaka, Dionisis Cavouras, Vassilia J. Sinanoglou

**Affiliations:** 1 Laboratory of Chemistry, Analysis & Design of Food Processes, Department of Food Science and Technology, University of West Attica, Agiou Spyridonos, 12243 Egaleo, Greece; gladika@uniwa.gr (G.L.); estrati@uniwa.gr (I.F.S.); tsiakath@uniwa.gr (T.T.); 2 Department of Biomedical Engineering, University of West Attica, Agiou Spyridonos, 12243 Egaleo, Greece; cavouras@uniwa.gr

**Keywords:** strawberry, shelf-life, image analysis, physicochemical properties, ATR-FTIR spectroscopy, antioxidant activity, antiradical activity, prediction model

## Abstract

The aim of the present study was to evaluate Marisol strawberries’ (*Fragaria* × *ananassa*) physicochemical quality and shelf-life during storage, using an integrated analytical approach. More specifically, the research aimed to assess the strawberries’ color, texture, and nutritional quality, over an 11-day storage period, employing physicochemical analyses, spectrophotometric assays, Attenuated Total Reflection-Fourier Transform Infrared (ATR-FTIR) spectroscopy, image analysis, and statistical tools. The results revealed significant changes in the outer surface texture and color characteristics, indicating spoilage progression. Physicochemical parameters such as water activity, moisture content, soluble solids, titratable acidity, and ascorbic acid content exhibited significant alterations, influencing the taste profile and freshness perception. Antioxidant and antiradical activities showed fluctuations, suggesting a potential decrease in phenolic content during storage. Moreover, the ATR-FTIR spectra findings confirmed the results regarding the moisture content, soluble solids, and total phenolic content. The integration of physicochemical and image analysis-derived features through a principal component analysis (PCA) enabled the accurate classification of samples based on storage days. Regression analysis, using these features, successfully predicted the storage day with high accuracy. Overall, this integrated analytical approach provided valuable information on the estimation of Marisol strawberries’ shelf-life and the prediction of their quality deterioration, contributing to better fruit management and the minimization of discards.

## 1. Introduction

The strawberry (*Fragaria × ananassa*) is a popular fruit with remarkable commercial influence mainly due to its health benefits and pleasing taste [1]. Taxonomically, it belongs to the *Fragaria* L. genus and the *Rosaceae* family [2]. It is recognized as a non-climacteric fruit, indicating that its ripening process is not significantly influenced by ethylene biosynthesis [3]. The strawberry’s yield of production and quality are both greatly influenced by environmental factors [4]. While the growth period of strawberries is limited, innovative farming techniques like hydroponics have been developed to enable year-round cultivation [5].

The strawberry is considered to be a functional food due to its rich content in bioactive constituents, which are related to antioxidant, antihypertensive, anti-inflammatory, antihyperlipidemic, antiproliferative, and other activities [6,7]. Several compounds, such as vitamins (ascorbic acid and folic acid), minerals, and phytochemicals including polyphenols, flavonoids, anthocyanins, and tannins, are responsible for most of these effects and are contained in high quantities in strawberries [8]. As far as the antioxidant activity of strawberries is concerned, it is mainly attributed to strawberries’ phenolic constituents and vitamin C [9].

Strawberries are well-known for their perishable nature, which leads to a very short shelf-life. Their short postharvest life can be attributed to several factors, such as a high sensitivity to mechanical injuries, high metabolic rates of this type of fruit, the presence of pathogenic microorganisms, and an increased loss of tissue turgidity over time. All these factors contribute to the rapid deterioration and loss of firmness and overall quality of the fruit and lead to high levels of strawberry waste on both the retail and consumer levels [10,11]. However, it is important to note that both the chemical composition and quality attributes of strawberries vary significantly due to the genotype, the cultivation techniques and geographic location of cultivation, the maturity stage at the time of harvest, as well as the pre- and postharvest treatments applied, and the storage conditions [12].

Considering the important role of strawberries in the fruit industry, several studies [10,13,14,15,16,17,18,19,20,21,22,23] have been conducted, including shelf-life studies on different varieties, maturity stages, or storage conditions and cultivation techniques. In these studies, a wide range of analytical techniques and methods have been used, including spectroscopic and chromatographic methods, sensory analysis, spectrophotometric assays, textural analysis, determination of sugars, organic acids, vitamins, and carotenoids, color evaluation, RNA extraction, etc. 

However, a single methodological approach is not capable of capturing the full spectrum of information required for a holistic evaluation of strawberries’ shelf-life, and limited research has been conducted on samples grown under hydroponic conditions during winter periods. Therefore, it is crucial to employ a more comprehensive approach by integrating multiple results obtained through several methods. 

In response to these knowledge gaps, our research combines a diverse set of analytical methods, both destructive and non-destructive, along with statistical analysis techniques, allowing us to establish meaningful relationships among these distinct analytical methodologies and providing a more comprehensive insight into the quality evolution of strawberries during storage. The main objective of this study was to evaluate the shelf-life of Marisol strawberries throughout storage using a comprehensive approach that included physicochemical analyses, spectrophotometric assays, Attenuated Total Reflection-Fourier Transform Infrared (ATR-FTIR) spectroscopy, image analysis, and statistical methods. In more detail, important quality parameters were quantified, such as moisture content, water activity, color parameters, total soluble solids’ content, textural attributes, titratable acidity (%), ascorbic acid content (%), total phenolic content, as well as antioxidant and antiradical activity. The results obtained from all these methods were then comparatively analyzed to evaluate the strawberries’ storage life under controlled temperature and humidity. Furthermore, statistical tools, like discriminant and regression analysis, were utilized to recognize significant patterns and correlations, and predictive models were developed based on the collected data to predict the shelf-life and fruit quality parameters during storage.

## 2. Materials and Methods

### 2.1. Strawberry Samples

This study was conducted on winter-grown strawberry samples of the Marisol variety (*Fragaria* sp., cv Marisol) that had been cultivated using hydroponic agriculture techniques in Greece. The strawberries samples (total 7.5 kg in boxes of 500 g each) were harvested at the point of commercial maturity by “K & K GREEN FARMS” (https://kkgreenfarms.gr/, accessed on 5 December 2023) and immediately transported in cold conditions to the laboratory, to prevent postharvest damage and spoilage, a day after being harvested, which is referred to as day 1. The strawberries were immediately stored in the laboratory’s cooled incubators (POL-EKO Cooled incubator ST 3, POL-EKO-APARATURA) under controlled temperature (8.0 ± 0.5 °C) and relative humidity (60 ± 2%) conditions until they were unfit for human consumption. The defective ones were eliminated prior to the treatment. To study the shelf-life of the fruits, representative samples were collected from the storage at regular intervals of 2–3 days, spanning a total period of 11 days. For all the measurements, 12 replicates of strawberry samples were randomly chosen from the incubators, and the shelf-life evaluations of the strawberries were conducted at the time points of 1, 4, 6, 8, and 11 days of storage. 

### 2.2. Image Analysis

**Figure 1:**
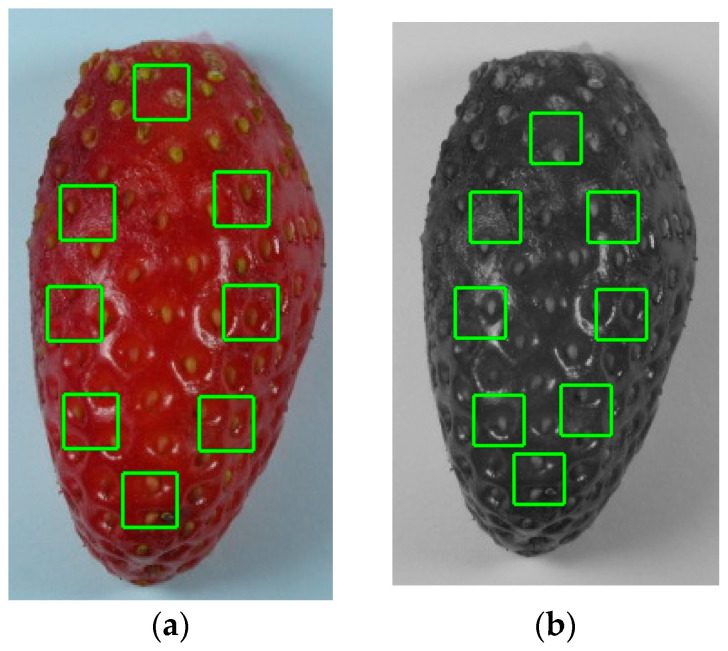
(**a**) Colored image of a strawberry sample, (**b**) grayscale version of the strawberry image used for textural feature calculation, and ROIs’ extraction indicated by the green boxes.

According to Zhang et al. [24], the color and textural features of a fruit can offer valuable insights into its quality or ripeness. In our study, the image analysis’ purpose was to examine the differences in the color and texture features of the strawberries during storage. The strawberries were photographed on both their outer and inner surface using a Sony DSCW800/B digital camera (IXUS 100 IS) from a distance of 15 cm, under fixed lighting conditions. The images were taken at a lens aperture of f = 4.6 and were saved in a JPEG format at a resolution of 1280 × 720 pixels. Eight regions of interest (ROIs) of 15 × 15 pixels each were selected from different areas on each strawberry sample. From the multiple ROIs of each photograph (Figure 1), eighteen textural features were extracted using a special-designed software. From the colored images, three color parameters (L*, a*, and b*) were extracted, using the rgb2lab function of Python’s skimage library. From the grayscale versions of the images, fifteen features were calculated according to Sinanoglou et al. [25]. These features were used to estimate and evaluate the color and textural changes that occur as a strawberry ages. 

### 2.3. Physicochemical Measurements during Strawberries’ Shelf-Life

The water activity of the strawberry samples was determined using an AquaLab dew point water activity meter (model 4TE, METERGroup, Inc., Pullman, WA, USA). 

The moisture levels of the strawberry samples were evaluated using an electronic moisture analyzer, specifically the Kern MLS 50-3 from KERN & SOHN GmbH, Balingen, Germany. Each strawberry sample, weighing between 0.2 and 0.4 g, was placed on a sample pan, and the moisture content was determined as a percentage by measuring the weight loss as the sample was dried using a halogen dryer that is embedded within the analyzer itself. The maximum temperature maintained throughout the measurement process was 120 °C.

To determine the total soluble solids (TSS) of the strawberry samples, a portable refractometer, specifically the Kern Optics Analogue Brix Refractometer ORA 80BB from KERN & SOHN GmbH in Balingen, Germany, was utilized. The strawberry samples were blended, and the produced juices were placed onto the prism of the refractometer. The TSS were obtained in Brix degrees (°Brix) [25].

The hue (h) angle in degrees of the strawberry samples were measured using a tristimulus chromatometer, the CR-400, from Minolta, Tokyo, Japan. The instrument was calibrated with a standard white plate (L*: 97.83, a*: −0.45, b*: +1.88). Measurements were taken from both the inner and outer surface of the strawberry samples.

The texture of the strawberry samples was evaluated using the texture analyzer TA-XTplusC by Stable Micro Systems, Godalming, UK, following the methodology described by Giannakourou et al. [26]. The textural attributes of firmness, cohesiveness, adhesiveness, springiness, and chewiness were calculated.

The titratable acidity (%) of the strawberry samples was determined using the AOAC (2012) method. The results were given as g of citric acid per 100 g of fruit, as citric acid is the primary organic acid present in strawberries.

The ascorbic acid content in the strawberry samples was measured using the indophenols titration method [27]. DCPIP (2,6-dichlorophenolindophenol) is an indicator dye that is reduced to a colorless form reacting with ascorbic acid, which is the reducing agent. The oxalic acid was used as a standard to determine the concentration of ascorbic acid in the fruit sample. The method involved adding a known amount of oxalic acid to the homogenized fruit sample to oxidize the ascorbic acid. The oxidized ascorbic acid was then titrated with a solution of DCPIP until the blue color of the dye disappeared, indicating that all the ascorbic acid had been oxidized. The amount of DCPIP solution used in the titration was used to calculate the concentration of ascorbic acid in the sample.

For each day of analysis, twelve replicates were randomly chosen from different strawberry packaging boxes for each measurement.

### 2.4. Phenolic Compound Extraction and Analysis Using Spectrophotometric Methods 

For the extract preparation, approximately 1 g of strawberry was mixed with a solution of aqueous methanol (80% *v*/*v*) in a ratio of the raw material to the solution of 1:5 (*w*/*v*). The resulting mixture was stored in sealed containers at 20 °C for 24 h. Afterwards, the extracts were filtered using a Buchner funnel, and the filtered liquid was further diluted in 10.00 mL of aqueous methanol. The extracts were then stored at 4 °C for spectrophotometric analysis. The spectrophotometric measurements were carried out in a Spectro 23, Digital Spectrophotometer (Labomed, Inc., Los Angeles, CA, USA), with each measurement being performed three times for accuracy. 

The total phenolic content (TPC) of the strawberry extracts was determined using a modified assay of the Folin–Ciocalteu method [28]. The measurements were taken at a wavelength of 750 nm, and the results were expressed as mg of gallic acid equivalents (GAE) per g of strawberry. To assess the antiradical activity of the strawberry extracts, the ABTS^•+^ radical assay was used [29], and the absorbance was measured at 734 nm. The antiradical activity was expressed as mg of Trolox Equivalents (TE) per g of strawberry. The Ferric Reducing Antioxidant Power (FRAP) assay was conducted to assess the antioxidant activity of the strawberry extracts [30], and the absorbance was measured at 595 nm. The results were expressed as mg of Fe (II) per g of strawberry.

### 2.5. LC-ESI(+)-MS/MS Analysis

A Liquid Chromatography Electrospray Ionization Tandem Mass Spectrometric analysis (LC-ESI(+)-MS/MS) was applied on the strawberry’s extracts, using a method developed in previous works by our group, as explicitly described by Tsiaka et al. [31]. The LC-MS/MS data were obtained in both a positive and a negative ionization mode by employing the same LC and MS conditions. The LC-MS/MS analysis was conducted using an Agilent 1200 HPLC system (Agilent Technologies, Santa Clara, CA, USA). The chromatographic separation utilized an Agilent Eclipse Plus C-18 reversed-phase column (50 mm × 2.1 mm inner diameter, 3.5 µm particle size) with an RRLC in-line filter kit (2.1 mm, 0.2 µm filter). The mobile phase consisted of water −0.2% *v*/*v* formic acid (Solvent A) and acetonitrile −0.1% *v*/*v* formic acid (Solvent B). The MS System used contained a 3200 Q TRAP triple-quadrupole linear ion trap mass spectrometer (Sciex, Framingham, MA, USA). All the spectra were processed using the Analyst Software, version 1.6, (AB SCIEX, Framingham, MA, USA).

### 2.6. Fourier Transform Infrared Spectroscopy with Attenuated Total Reflectance (ATR-FTIR)

The strawberry samples were evaluated via Fourier Transform Infrared Spectroscopy with Attenuated Total Reflectance (ATR-FTIR), at room temperature, using an ATR spectrometer (Shimadzu, IRAffinity-1S FTIR Spectrometer, Kyoto, Japan). The inner strawberry flesh without pericarp was used and was placed in the FTIR spectrometer. The data obtained from this analysis were then processed and analyzed using the LabSolutions IR software (version 2.21) [32].

### 2.7. Discriminant Analysis

In order to discriminate strawberries of different storage days, the textural and color features extracted from the images of the samples as well as the results of the spectrophotometric assays were subjected to machine learning (ML) methods. Ten (10) different ML-classifiers from the scikit-learn library of the Python programming language (https://scikit-learn.org/, accessed on 5 November 2023) were tested on the five (5) classes formed by the five days of measurements, and, as described in detail by Sinanoglou et al. [25], the ML-system of the highest precision in classifying the strawberry ROIs to the correct storage day was determined. The scikit-learn library’s 10-ML algorithms employed were the following: Nearest Centroid, Naïve Bayesian, K-Nearest Neighbor, Linear Discriminant Analysis, Logistic Regression, Perceptron, Multi-Layer Perceptron, Random Forest, Classification and Regression Decision-Tree, and Support Vector Machines. For our data, the best-performing classifier was the Random Forest classifier. Each combination of features was condensed into two components called PCA1 and PCA2 using principal component analysis (PCA), and the results of the discriminant analysis, evaluated using Python’s scikit-learn library’s ‘RepeatedKFold’ function and a ML-classifier, were visualized by generating two-dimensional scatter plots based on the PCA, with class-separating surfaces to highlight the distinctions between different classes.

### 2.8. Statistical Analysis

The statistical analysis of the image and physicochemical analysis’ results during storage was accomplished through a non-parametric Mann–Whitney–Wilcoxon test for the two classes, implemented through Python’s scipy.stats library (https://docs.scipy.org/doc/scipy/tutorial/stats.html, accessed on 5 November 2023). 

The ATR-FTIR results were analyzed with a one-way ANOVA and a post hoc analysis in SPSS (IBM SPSS Statistics, version 29.0, Chicago, IL, USA), with a significance level of 95% (*p <* 0.05). 

Moreover, multiple regression was employed using the Gradient-Boosting Regressor model and the K-fold evaluation method to predict the actual storage days of the strawberries via the images extracted and the physicochemical features.

## 3. Results and Discussion

### 3.1. Textural-Image Analysis of Strawberries’ Outer Surface during Storage

In order to assess the storage effect on the strawberries, the textural features extracted from the grayscale images of the fruit’s outer surface were subjected to machine learning methods. 

**Figure 2:**
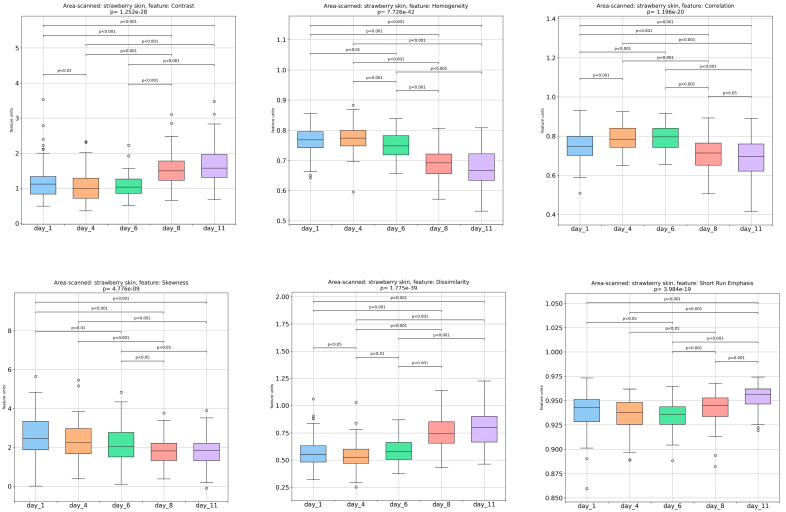

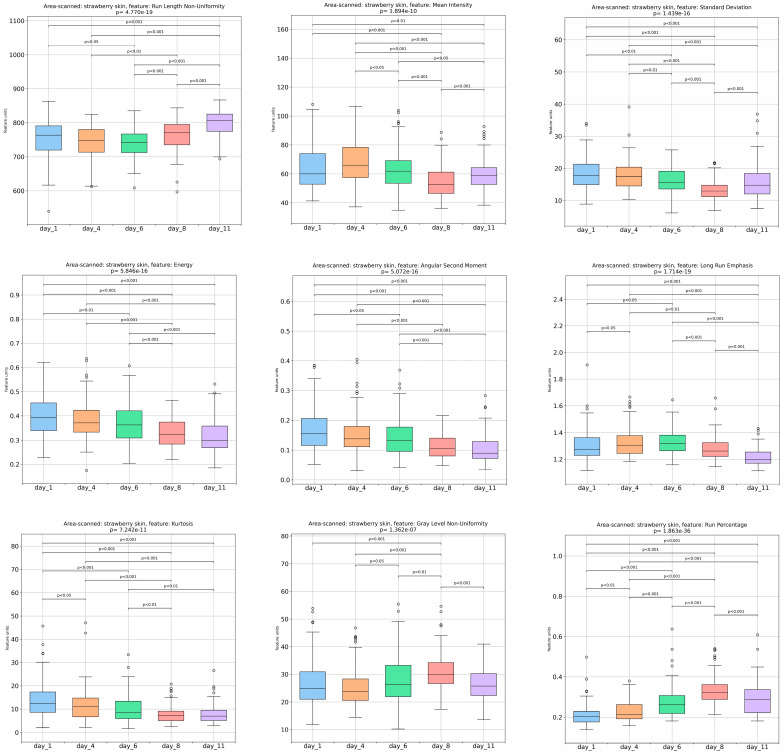
Fluctuations and p-values between days in image analysis-derived features (contrast, homogeneity, correlation, skewness, dissimilarity, short-run emphasis (SRE), run length non-uniformity (RLN), mean, standard deviation, energy, angular second moment (ASM), long-run emphasis (LRE), kurtosis, gray level non-uniformity (GLN), and run percentage (RP)) of the outer surface of strawberries over storage periods of 1, 4, 6, 8, and 11 days.

The fluctuations in the image analysis’ computed features of the strawberries’ outer surface, extracted in shades of gray, are presented in Figure 2. Based on the results, a significant increase was observed in the contrast values (intensity differentiations in the image) along with a decrease in the homogeneity (the distribution proximity of image elements) and correlation (gray-tone linear-dependencies in the image) values [24]. These variations became more pronounced from the sixth day until the final day of storage, suggesting that the texture of the outer surface of the strawberries became more diverse, while the smoother areas decreased. The skewness exhibited a gradual decrease during strawberry storage, which was especially significant on day 8. According to Takemoto et al. [33], the skewness of the luminance distribution can be used as an indicator of the perceived freshness of strawberries. Therefore, this finding could suggest that the shine or smoothness of the surface of the strawberries decreased over storage time. Moreover, the progressive increase in dissimilarity (variations in image texture), short-run emphasis (small formations with equal gray levels), and run length non-uniformity (unevenness in image structures) indicated the rise in heterogeneity on the strawberries’ surface. These changes in the surface characteristics of the strawberries observed during storage may suggest that the fruits were undergoing spoilage. More specifically, the short-run emphasis values increased noticeably from day 8 to day 11. A possible interpretation of this change is the predominance of the finer textures of strawberry. Moreover, the term run length non-uniformity exhibited a noteworthy (*p <* 0.001) increase from day 6 to day 11, which could suggest an increased variability in the lengths of the uniform regions of strawberry texture during storage.

In addition, a gradual and significant decrease was observed in the values of the mean (average of the intensities from the pixels of all images), standard deviation (image gray levels’ variation from the mean value), energy (homogeneity in the image’s gray levels), angular second moment (original image’s homogeneity), and long-run emphasis (large formations of equal gray levels), until day 11 of the experiment. The decrease in angular second moment suggests a reduction in the uniformity of gray intensity distribution, resulting in a less uniform texture of the fruit. Additionally, the significant decrease in long-run emphasis, from day 6 to day 11, implies a decrease in homogeneity, roughness, and long-range spatial correlations (the texture becomes less correlated across long distances). This results in a reduction in the repeating patterns aligned along a specific direction. 

The gray level non-uniformity values, which measure the variability in the gray levels within an image, resulted in significant variations during storage. On the other hand, the run percentage, which indicates the overall homogeneity of the histogram, increased significantly (*p <* 0.001) from day 1 to day 8 and then decreased until the last day of storage. This suggests that the strawberries had a finer texture during that period. 

### 3.2. Image Analysis-Extracted Color Parameters of Strawberries’ Outer and Inner Surface

**Figure 3:**
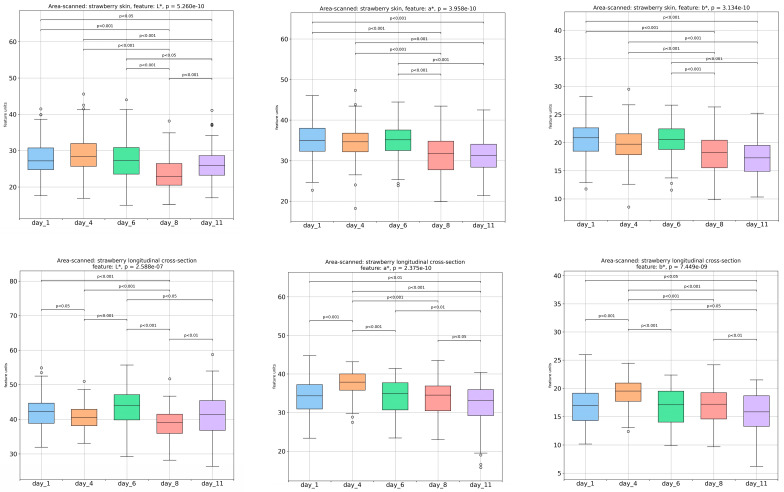
Image analysis-derived colored features (lightness L*, a* parameter, b* parameter) of both the outer (skin) and inner surface of strawberries over a storage period of 1, 4, 6, 8, and 11 days.

The variations in the color parameters L* (indicates the lightness), a* (negative values correspond to green, while the positive values correspond to red shades), and b* (negative values signify blue shades, while the positive values indicate yellow shades) extracted from the colored images of both the outer and inner samples’ surfaces are presented in Figure 3. All the color parameters of the outer surface revealed a significant reduction from day 6 to day 8 and then a* and b* stabilized until day 11, whereas L* increased (*p <* 0.001). According to Falah et al. [14], the reduction in color parameters’ values can be associated with a decrease in anthocyanin concentration, flavonoid and phenolic concentration, as well as the total antioxidant capacity of the fruit, during storage. As far as the inner surface of the samples is concerned, the a* and b* parameters showed a significant (*p <* 0.001) increase on day 4, after which point they decreased gradually until day 11, while the L* parameter displayed significant variations which resulted in an overall decrease on day 11.

### 3.3. Storage Effect on the Physicochemical Features of Strawberries

During the 11-day storage period at 8.0 ± 0.5 °C, various physical and chemical characteristics of the strawberry samples were assessed. This included the determination of water activity (aw), moisture content (%), total soluble solids (TSS), titratable acidity (%), and ascorbic acid content (%).

**Figure 4:**
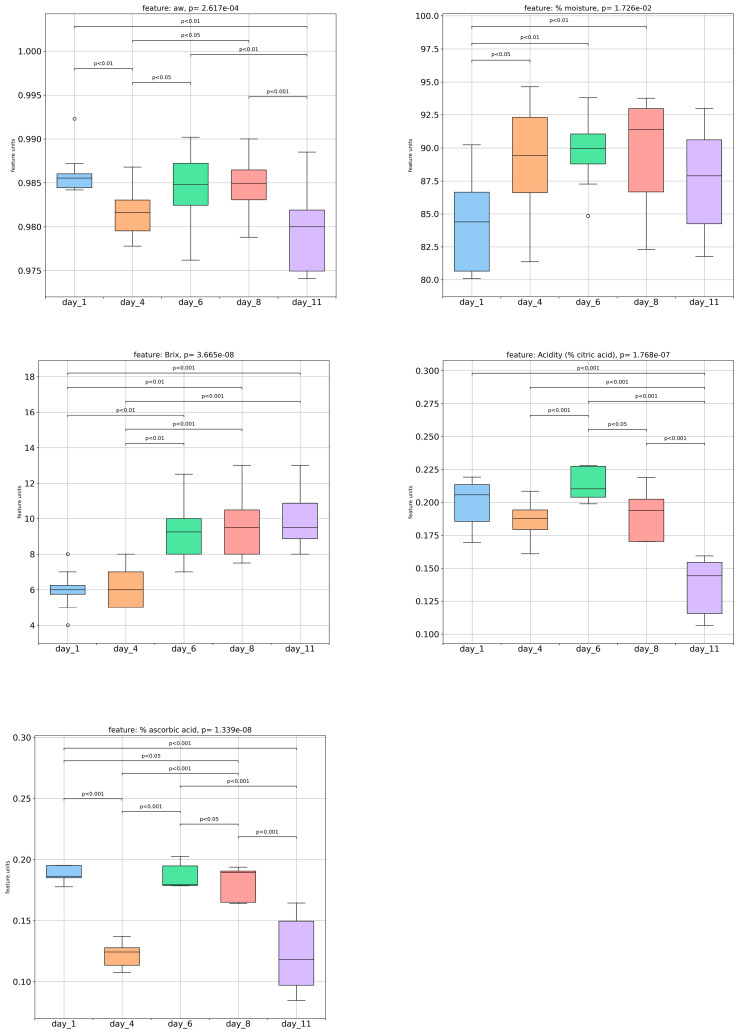
Analysis of water activity, moisture content, Brix, titratable acidity, and ascorbic acid content of the strawberry samples during storage at 8.0 ± 0.5 °C.

Throughout the storage period, significant fluctuations were observed in the water activity levels until day 6, which then plateaued until day 8 and subsequently decreased until day 11 (Figure 4). On the other hand, the moisture content increased progressively until day 8, followed by an insignificant decrease until day 11 (Figure 4). Although one of the major factors causing the high susceptibility of strawberries to spoilage is fast water loss [16], this phenomenon was effectively minimized by the storage conditions implemented in this research. The ripening and aging process of strawberries occurs quickly due to their high respiration rate, which leads to a softening of the skin texture caused by a weak epidermis, especially when stored at room temperature [14]. However, the maintenance of fresh strawberries at a low and controlled temperature, such as in a refrigerated environment, has been shown to be an effective way to prolong their shelf-life [14]. The total soluble solids (TSS) content remained relatively unchanged until the fourth day, then demonstrated a notable (*p <* 0.05) increase from day 4 to day 6, and then remained stable until the end of storage. The total soluble solids’ values can be correlated with the sugar content, as carbohydrates are the major substances that influence TSS values. So, it was assumed that this significant increase in carbohydrates from day 4 to day 6 could be associated with the hydrolysis of polysaccharides to simpler sugars. Moreover, the total soluble solids (TSS) increase could be attributed to the conversion of sucrose to invert sugars through hydrolysis during storage [20]. 

The titratable acidity (TA) did not show significant changes until the fourth day of storage (Figure 4). After that, the TA increased meaningfully from day 4 to day 6 and then gradually decreased and reached its lowest value on the eleventh day. Similar results were reported by Jouki and Dadashpour [16] in strawberries stored at 4 °C. It is suggested that the decrease in TA may be due to acid oxidation during Krebs’ cycle or to other metabolic pathways occurring during the ripening process [10,16,34]. Apart from factors such as texture, aroma, and color, which can influence the overall perception of the taste of strawberries, the TA and TSS can be used as quality indicators for assessing strawberries’ taste superiority. Rutkowski et al. [21] suggested that a maximum total acidity (TA) level of 0.8% and a minimum total soluble solids (TSS) level of 7% should be maintained in order to achieve an acceptable taste in strawberries. The strawberry samples analyzed in this research had acceptable acidity values during the whole storage period. In contrast, the total soluble solids fell into the acceptable range of the minimum value (7%) from day 6 until day 11. Therefore, the strawberry samples analyzed in this research had an acceptable taste profile from day 6 to day 11.

The ascorbic acid content, which was determined using the indophenols titration method, showed a significant decrease from day 1 to day 4, followed by a significant increase on day 6 of storage. After this variation, its value gradually decreased until the end of the storage period. According to Falah et al. [14], ascorbic acid is unstable and can be easily oxidized, particularly when stored at high temperatures. 

The increase in ascorbic acid levels may be attributed to the hydrolysis of ascorbic acid glycosides, specifically the 2-O-β-D-glucopyranosyl L-ascorbic acid type. This compound does not react with DCPIP (2,6-dichlorophenolindophenol), in contrast to the free form of ascorbic acid. This hypothesis may be confirmed by Richardson et al. [35,36], who identified this particular derivative of vitamin C in fruits and leaves from the *Rosaceae* family. 

**Figure 5:**
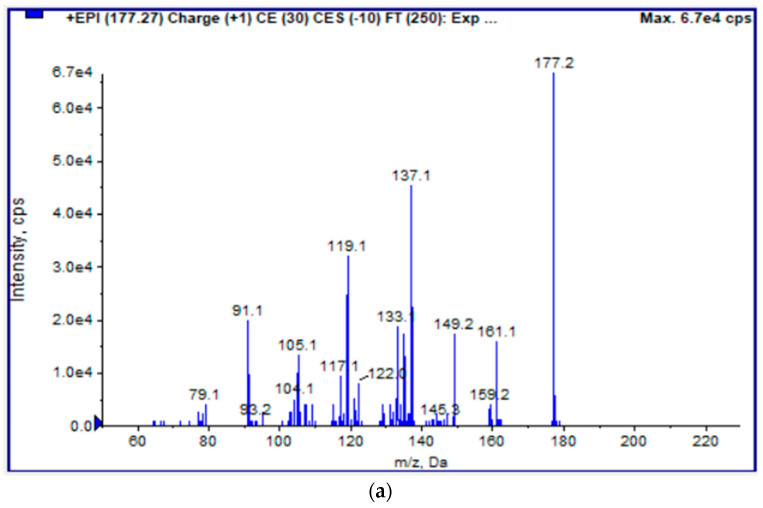

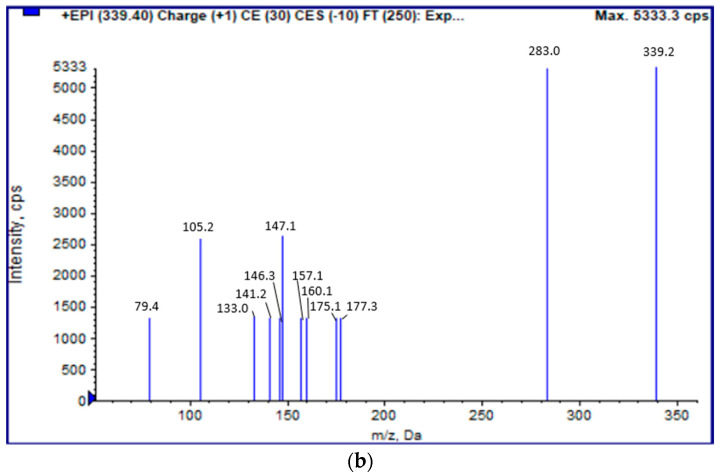
LC-MS/MS spectra assigned to (**a**) ascorbic acid and (**b**) 2-O-β-D-glucopyranosyl L-ascorbic acid.

In order to find evidence pointing to the presence of this particular glycoside in the strawberry samples, an LC-ESI(+)-MS/MS analysis was conducted. Richardson et al. [35] identified the ion [M-H]^−^ at a negative ionization, with a mass-to-charge ratio (*m*/*z*) of 337.0749, suggesting that it is the glycoside of ascorbic acid. Thus, the parent ion of the ascorbic acid glycoside in a positive ionization is expected at *m*/*z* [M-H]^+^ =339.2. Moreover, according to the Human Metabolome Database, HMDB (https://hmdb.ca/, accessed on 5 December 2023), the positive MS–MS spectrum of ascorbic acid exhibits characteristic fragments at 43.2282, 69.0007, 79.0618, 91.0591, 102.7396, 103.0542, 105.0684, 115.0569, 121.0628, 132.0598, 133.0745, 149.0596, and 177.0484. The fragmentation pattern of the *m*/*z* 177.2 ion (presented in Figure 5) was very similar to that of ascorbic acid. In addition, the observed *m*/*z* = 339.2 was tentatively assigned to the 2-O-β-D-glucopyranosyl L-ascorbic acid type, since the MS2 spectra of this *m*/*z* presented common ions and fragmentation pattern to those of ascorbic acid. However, due to the relatively low intensities of these fragments, further investigation, which could include the analysis of the compounds’ standard solutions and/or the use of high-resolution MS instrumentation, is required to conclusively verify and validate the presence of ascorbic acid’s glycoside and its potential impact on the ascorbic acid content of strawberries.

The hue angle (h) values of both the internal and external surface of the strawberry samples during storage were determined using a chromatometer and are presented in Table 1. The color appearance parameter h was relatively stable during the whole storage period on both the external and internal surface of the strawberry samples. Given the fact that a hue demonstrates the way in which someone perceives a color, based on the colors of the rainbow, it seems that consumers’ perception of a strawberry’s color could remain unchangeable for the entire storage period. 

During storage, the strawberries’ firmness (Table 2) decreased meaningfully (*p <* 0.05) between day 4 and day 6 and then remained relatively stable until the end of the storage period. This reduction may be attributed to the hydrolysis of polysaccharides that contribute to the structure of the fruit. The increase in sugars from day 4 to day 6, as determined by means of the total soluble solids’ analysis, supports this hypothesis. Additionally, Falah et al. [14] suggest that the deterioration in the texture of the fruit may be linked to a decrease in the turgor pressure exerted on the cell membrane and a reduction in the concentration of pectin during the fruit’s maturation process. Moreover, adhesiveness (the amount of force needed to detach a product’s surfaces from the probe) and cohesiveness (the ability of a food to maintain its structure during the first and second compression) demonstrated slight decreases and increases (*p >* 0.05), respectively, throughout the 11-day storage period, which became significant (*p <* 0.05) on day 11. 

### 3.4. Spectrophotometric Assays 

**Figure 6:**
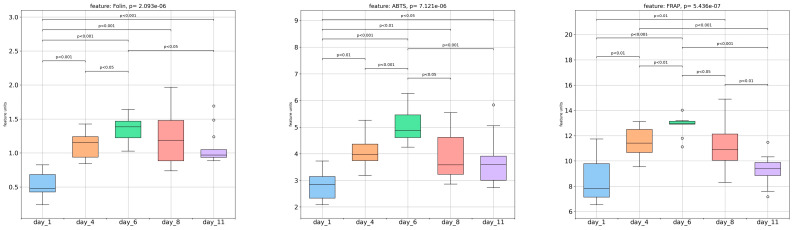
Total phenolic content (TPC), antiradical activity (ABTS^•+^), and antioxidant activity (FRAP) of the strawberry samples during storage at 8.0 ± 0.5 °C.

Figure 6 displays the changes in total phenolic content (TPC), antiradical activity (ABTS^●+^), and antioxidant activity (FRAP) over the storage period of the strawberries. Based on the findings, all three assays exhibited a significant and progressive increase in their values from day 1 to day 6, followed by a gradual reduction, not necessarily significant, until day 11. The TPC increase can be primarily attributed to the increase in anthocyanins, which continue to be synthesized even after harvest and under low temperatures or continue to be released from the membranes during cold storage [18,23]. The decrease in TPC and antiradical and antioxidant activity values observed from day 6 until the end of storage can be explained by the deterioration of cell structure as strawberries undergo senescence [10].

The results of the spectrophotometric assays conducted on the strawberry extracts revealed notable pairwise correlations (*p <* 0.05). Specifically, a high positive correlation of 0.86 was observed between the FRAP and ABTS^•+^ values. Furthermore, the ABTS^•+^ and TPC values as well as the TPC and FRAP values displayed strong positive correlations of 0.80 and 0.77, respectively.

### 3.5. Interpretation of ATR-FTIR (Attenuated Total Reflection-Fourier Transform Infrared) Spectra 

**Figure 7:**
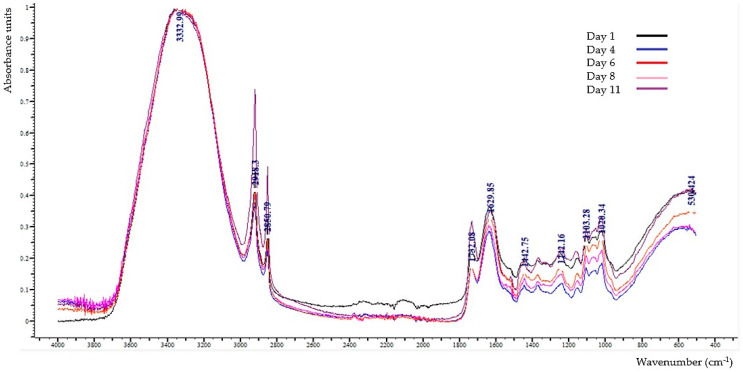
Overlay of the ATR-FTIR spectra obtained from 4000 to 499 cm^−1^ of the strawberries at 1, 4, 6, 8, and 11 days of storage.

The overlay of the ATR-FTIR spectra of the strawberries over the 11-day storage period is presented in Figure 7.

In detail, the absorption band at 3645–3600 cm^−1^ is assigned to the O-H stretching vibrations of the phenolic compounds [37]. The broad absorption band at 3380 cm^−1^ is related to the stretching of O-H that consists in water, organic acids (especially citric acid), carbohydrates, amino acids, as well as alcohols [38,39,40]. The bands at 2918–2920 cm^−1^ and 2850–2855 cm^−1^ are associated with the asymmetric and symmetric C-H stretching, respectively, of the CH_3_ and CH_2_ of carbohydrates and carboxylic and amino acids [38,39,41]. Furthermore, the band at 1730–1742 cm^−1^ is due to the C=O stretching found in compounds like ethyl hexanoate, methyl, and ethyl butanoate, affecting fruit fragrance [40]. In addition, according to Drobek et al. [3], the bands at 1742 cm^−1^ are related to the ester bonds. The band at 1632–1647 cm^−1^ is related to the bending vibrations of hydroxyl groups (O-H) that are characteristic of carbohydrates, water, phenols, and organic acids, which can also be related to the sample’s moisture content [38,39,40,42]. Moreover, the band at 1510–1520 cm^−1^ is assigned to the stretching of C=C-C in the aromatic ring structure [37]. The bands at 1420 cm^−1^ and 1457 cm^−1^ are due to the combination of O-H bending and C-H rocking vibrations and CH_2_ scissoring in monosaccharides, respectively [38,39,41,43]. The band at 1351–1378 cm^−1^ is associated with the presence of citric acid [44], while the band at 1245–1230 cm^−1^ is assigned to the C-O stretching present in carbohydrates, phenolics, di- and polysaccharides, as well as glycosylated anthocyanins [45]. Furthermore, the band at 1149–1155 cm^−1^ corresponds to the C-H deformation vibrations in carbohydrates [39]; the band at 1146–1147 cm^−1^ is due to the vibrations caused by the C-O-C glycosidic bond between uronic acids [46], and the band at 1105 cm^−1^ is associated with the C-O and C-C stretching in carbohydrates [47]. Generally, the area 1020–1100 cm^−1^ links to the characteristic peaks of the main sugars found in strawberry (glucose, fructose, and sucrose), while the bands at 1050–1055 cm^−1^ and 1022–1028 cm^−1^ are ascribed to sucrose and glucose, respectively [48,49,50]. The bands at 700–900 cm^−1^ are linked to the presence of nucleic acids [50], and the band at 720–750 cm^−1^ is attributed to rocking vibrations of the methylene group, (CH_2_)_n_ (*n* ≥ 3) [37]. The band at 610–680 cm^−1^ is related to the bending vibrations of alkyne C-H [37], while the band at 523 cm^−1^ is associated with the C-C-C and C-O-C in-plane bending in the glycosidic bond [43].

The intensities of the absorption bands in the strawberry samples’ spectra during their storage period are given in Table 3. The most interesting findings are listed below. 

The intensities of the peak at 3645–3600 cm^−1^, associated with the presence of phenolic compounds, showed significant fluctuations during storage, which were consistent with the variation in TPC values over storage time, except for day 6. The intensities at 2918–2920 cm^−1^ and 2850–2855 cm^−1^ exhibited an increase (*p <* 0.05) from day 4 to day 6, reaching their highest recorded values. The intensity at 1730–1742 cm^−1^ of the C=O in esters, probably influenced by the fruits’ fragrance, remained constant throughout the whole storage period. The peak observed at 1632–1647 cm^−1^, associated with the moisture content of the sample, demonstrated an overall increase, aligning with the results obtained from the moisture content analysis. The intensity at 1510–1520 cm^−1^ exhibited a statistically significant increase from day 4 to day 6, reaching its maximum value, followed by a significant decrease until day 8. These fluctuations are well-related to the TPC and antioxidant and antiradical activity variations, confirming the fact that phenolic compounds are the principal bioactive constituents of strawberries. The bands at 1050–1055 cm^−1^, most likely attributed to sucrose, and at 1022–1028 cm^−1^, which corresponds to the vibrations of the glucose pyranose ring, displayed no significant changes in intensity, except for a statistically significant (*p <* 0.05) increase on the last day (day 11) of storage, confirming the TSS increase at the end of the storage period.

### 3.6. Statistical Analysis

#### 3.6.1. Discriminant Analysis

**Figure 8:**
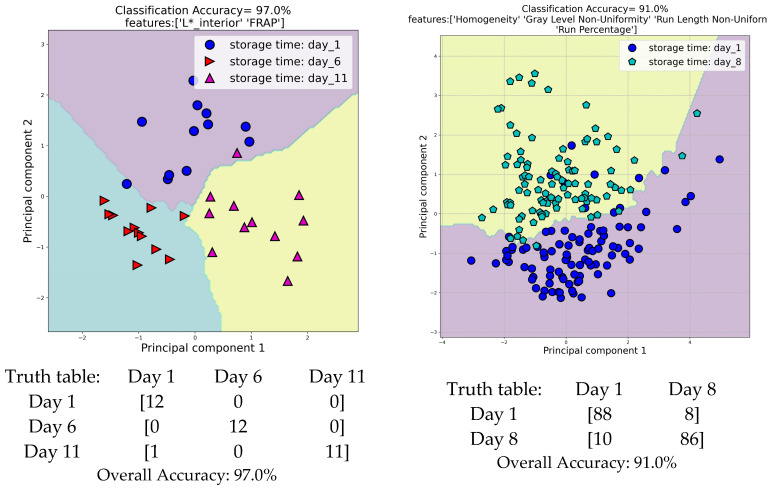
Discriminant analysis between storage days based on physicochemical and image-extracted features.

Machine learning algorithms were utilized to perform a discriminant analysis using both the physicochemical and image-extracted features. The objective was to identify the most suitable combinations of features that could effectively discriminate amongst different days of storage. In Figure 8, scatter diagrams based on a principal component analysis (PCA) illustrate the successful classification of the different storage days. The first scatter diagram demonstrates the optimal classification among days 1, 6, and 11 of storage, achieving an overall discrimination accuracy of 97.0%. This classification was accomplished using the feature L* (lightness) of the inner surface of the fruit and the values of the FRAP assay. In this classification, the designed high-performance machine learning system classified correctly all the samples from days 1 and 6 and eleven out of twelve samples from day 11. The second scatter diagram presents the best classification among days 1 and 8, using four textural features (homogeneity, gray level non-uniformity, run length non-uniformity, and run percentage) extracted from images of the outer surface of the strawberries. The overall accuracy of the classification of these days of storage was found to be 91.0%. In this classification, the designed machine learning system classified correctly 88 samples of day 1 and 86 samples of day 8 out of a total of 96 samples.

#### 3.6.2. Regression

**Figure 9:**
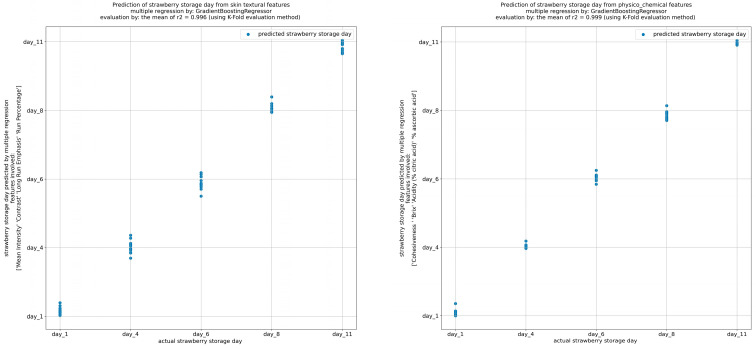
Prediction of storage days from skin textural and physicochemical features.

A regression analysis was conducted for the development of predictive models based on both physicochemical and image-extracted features, as presented in Figure 9. The primary objective of these models was to predict the storage day of the strawberries. For this purpose, the gradient-boosting regressor model was employed to predict the strawberry storage day, using the K-fold evaluation method, from the textural, image-extracted features. The textural features with the highest coefficient of determination (R^2^ = 0.996) where the mean value, the contrast (con), the long-run emphasis (LRE), and the run percentage (RP). In addition, using the same model, storage day was predicted through the cohesiveness, total soluble solid content (Brix), % acidity, and % ascorbic acid values, with R^2^ = 0.999.

## 4. Conclusions

In the current research, an integrated analytical approach, which combined the non-destructive method of image analysis with infrared spectroscopy, spectrophotometry, texture and color analysis, and the determination of characteristic components, was used, aiming to study strawberries shelf-life during storage. The most remarkable outcomes are summarized below.

Regarding the analysis of image-derived textural features of the outer surface of strawberries, the storage progress resulted in a decrease in the strawberries’ overall uniformity, an increase in their diversity, and in the emergence of finer textures, especially after the sixth day of storage. These changes indicate freshness deterioration and spoilage progression. Moreover, the redness (a* value) and yellowness (b* value) of the outer surface of the strawberries demonstrated a notable decrease from day 8 onwards, while the lightness (L* value) increased. Regarding the inner surface, the a* and b* parameters experienced a significant increase on day 4, followed by a gradual decrease until the last day of the experiment. In contrast, the L* parameter exhibited significant fluctuations, resulting in an overall decrease on day 11. The color appearance parameter hue (h) remained relatively stable over the entire storage period on both the outer and inner surfaces of the strawberry samples.

Regarding the physicochemical characteristics, the strawberries’ water activity subsequently decreased during storage. The total soluble solids values (Brix) displayed a significant increase on day 6, stabilizing thereafter, until the end of the storage period. Concurrently, the titratable acidity (TA) of the samples showed a significant increase on day 6, followed by a gradual decrease, reaching its lowest value on the eleventh day. Based on these results, the taste profile of the strawberry samples remained acceptable from day 6 to day 11. The ascorbic acid content showed a notable decrease on day 4, followed by a significant increase on day 6, which was potentially attributed to the presence of ascorbic acid glycosides. After this fluctuation, the ascorbic acid content gradually decreased until the end of the storage period. The texture attributes showed mostly non-significant variations, suggesting that the strawberries’ firmness and cohesiveness were not degraded during storage. The results of the TPC and of the antioxidant and antiradical activity provided strong correlations among them, reaching their highest values on day 6. The results of the ATR-FTIR interpretation confirmed many of the findings of the physicochemical analysis, such as the total phenolic content, total soluble solids, and moisture content fluctuations. The high-accuracy classification of the strawberry samples among different days of storage, based on both physicochemical and image-extracted features, was achieved using a PCA analysis. Furthermore, a regression analysis was applied in order to predict the day of storage of the strawberry samples, as a tool for estimating their shelf-life and quality. These models were trained using both physicochemical and image-extracted features and achieved a high accuracy prediction of the actual storage day.

Our current study, focused on both destructive and non-destructive methods, provided valuable foundational insights. However, an increase in sampling size would be pivotal in enriching our dataset and deliver a valid and robust methodology capable of accurately predicting the shelf-life of strawberries. To take this a step further, special focus should be given to the elucidation and quantification of phytochemicals that may serve as biomarkers of strawberry quality during storage. As a next step, we plan to focus exclusively on non-destructive methods, informed by the conclusions of this study, aiming to improve our analysis and contribute to advancements in assessing strawberries’ shelf-life.

## Figures and Tables

**Table 1:** Hue angle (h) of the strawberry samples during storage at 8.0 ± 0.5 °C.

Days	h (Internal Surface)	h (External Surface)
1	46.62 ± 2.04	24.86 ± 3. 40
4	44.45 ± 1.68	26.60 ± 4.46
6	45.70 ± 1.61	25.74 ± 4.19
8	45.36 ± 0.70	26.81 ± 4.71
11	44.26 ± 6.74	25.26 ± 2.38

Values in the same column do not present significant differences (*p >* 0.05).

**Table 2:** Firmness (N), adhesiveness, and cohesiveness of the strawberry samples during storage at 8.0 ± 0.5 °C.

Days	Firmness	Adhesiveness	Cohesiveness
1	4.70 ± 1.66 ab ^1^	−0.024 ± 0.018 a	0.26 ± 0.03 a
4	6.40 ± 1.91 a	−0.023 ± 0.013 a	0.30 ± 0.04 ab
6	4.24 ± 1.17 b	−0.039 ± 0.013 ab	0.25 ± 0.04 a
8	4.88 ± 1.20 ab	−0.031 ± 0.015 ab	0.27 ± 0.03 a
11	5.07 ± 1.44 ab	−0.044 ± 0.019 b	0.34 ± 0.10 b

^1^ Different letters in the same column indicate a significant difference (*p <* 0.05).

**Table 3:** Mean intensities of the ATR-FTIR spectral absorbance bands of the strawberry samples during storage.

Regions (cm^−1^)	Day 1	Day 4	Day 6	Day 8	Day 11
3645–3600	-	0.020 ± 0.003 b ^1^	0.011 ± 0.003 c	0.023 ± 0.009 b	0.011 ± 0.003 c
3380	0.006 ± 0.001 ac	0.004 ± 0.002 b	0.007 ± 0.001 c	0.006 ± 0.001 ac	0.005 ± 0.001 ab
2918–2920	0.317 ± 0.040 ab	0.251 ± 0.070 a	0.370 ± 0.063 b	0.292 ± 0.072 a	0.279 ± 0.062 a
2850–2855	0.181 ± 0.030 ab	0.134 ± 0.048 a	0.210 ± 0.053 b	0.174 ± 0.040 ab	0.202 ± 0.038 b
1730–1742	0.111 ± 0.014 a	0.100 ± 0.021 a	0.109 ± 0.030 a	0.104 ± 0.017 a	0.093 ± 0.016 a
1632–1647	0.171 ± 0.012 a	0.184 ± 0.012 ab	0.188 ± 0.010 ab	0.197 ± 0.023 b	0.190 ± 0.013 b
1510–1520	0.010 ± 0.005 ac	0.007 ± 0.003 c	0.017 ± 0.006 b	0.012 ± 0.004 a	0.011 ± 0.003 a
1420–1457	0.020 ± 0.006 a	0.019 ± 0.006 a	0.012 ± 0.003 b	0.017 ± 0.005 ab	0.019 ± 0.004 a
1351–1378	0.017 ± 0.004 a	0.006 ± 0.004 b	0.018 ± 0.004 a	0.015 ± 0.005 a	0.021 ± 0.003 a
1245–1230	0.046 ± 0.006 a	0.035 ± 0.007 b	0.047 ± 0.012 a	0.043 ± 0.014 ab	0.054 ± 0.009 a
1149–1155	0.023 ± 0.009 a	0.021 ± 0.008 a	0.026 ± 0.004 a	0.020 ± 0.010 a	0.022 ± 0.011 a
1105	0.058 ± 0.011 a	0.052 ± 0.012 a	0.054 ± 0.007 a	0.053 ± 0.009 a	0.056 ± 0.010 a
1050–1055	0.011 ± 0.003 a	0.006 ± 0.003 b	0.011 ± 0.004 a	0.008 ± 0.001 ab	0.015 ± 0.002 c
1022–1028	0.068 ± 0.013 ab	0.054 ± 0.013 a	0.060 ± 0.015 a	0.064 ± 0.018 a	0.081 ± 0.008 b
750–720	0.004 ± 0.002 a	0.003 ± 0.001 ab	0.002 ± 0.001 b	0.003 ± 0.001 ab	0.002 ± 0.001 b
680–610	0.001 ± 0.001 a	0.002 ± 0.002 a	0.001 ± 0.003 a	0.001 ± 0.001 a	-
523	0.009 ± 0.002 a	0.010 ± 0.003 a	0.010 ± 0.003 a	0.009 ± 0.002 a	0.008 ± 0.002 a

^1^ Different letters in the same row show significant differences (*p <* 0.05).

## Data Availability

All the data presented in this study are available within the article.

## References

[1] Warner R., Wu B.-S., MacPherson S., Lefsrud M. (2021). A Review of Strawberry Photobiology and Fruit Flavonoids in Controlled Environments. Front. Plant Sci..

[2] Azam M., Ejaz S., Naveed Ur Rehman R., Khan M., Qadri R., Asao T., Asaduzzaman M. (2019). Postharvest Quality Management of Strawberries. Strawberry-Pre-and Post-Harvest Management Techniques for Higher Fruit Quality.

[3] Drobek M., Frąc M., Zdunek A., Cybulska J. (2020). The Effect of Cultivation Method of Strawberry (*Fragaria* × *ananassa* Duch.) cv. Honeoye on Structure and Degradation Dynamics of Pectin during Cold Storage. Molecules.

[4] Khammayom N., Maruyama N., Chaichana C. (2022). The Effect of Climatic Parameters on Strawberry Production in a Small Walk-In Greenhouse. AgriEngineering.

[5] Sreedevi T.R., Santosh Kumar M.B. Digital Twin in Smart Farming: A Categorical Literature Review and Exploring Possibilities in Hydroponics. Proceedings of the 2020 Advanced Computing and Communication Technologies for High Performance Applications (ACCTHPA).

[6] Basu A., Nguyen A., Betts N.M., Lyons T.J. (2014). Strawberry As a Functional Food: An Evidence-Based Review. Crit. Rev. Food Sci. Nutr..

[7] Giampieri F., Alvarez-Suarez J.M., Battino M. (2014). Strawberry and Human Health: Effects beyond Antioxidant Activity. J. Agric. Food Chem..

[8] Afrin S., Gasparrini M., Forbes-Hernandez T.Y., Reboredo-Rodriguez P., Mezzetti B., Varela-López A., Giampieri F., Battino M. (2016). Promising Health Benefits of the Strawberry: A Focus on Clinical Studies. J. Agric. Food Chem..

[9] Hernández-Martínez N.R., Blanchard C., Wells D., Salazar-Gutiérrez M.R. (2023). Current State and Future Perspectives of Commercial Strawberry Production: A Review. Sci. Hortic..

[10] Gol N.B., Patel P.R., Rao T.V.R. (2013). Improvement of Quality and Shelf-Life of Strawberries with Edible Coatings Enriched with Chitosan. Postharvest Biol. Technol..

[11] Kelly K., Madden R., Emond J.P., Do Nascimento Nunes M.C. (2019). A Novel Approach to Determine the Impact Level of Each Step along the Supply Chain on Strawberry Quality. Postharvest Biol. Technol..

[12] Bhat R., Stamminger R. (2015). Preserving Strawberry Quality by Employing Novel Food Preservation and Processing Techniques—Recent Updates and Future Scope—An Overview: Preservation and Processing Techniques for Strawberries. J. Food Process Eng..

[13] Cordenunsi B.R., Genovese M.I., Oliveira Do Nascimento J.R., Aymoto Hassimotto N.M., José Dos Santos R., Lajolo F.M. (2005). Effects of Temperature on the Chemical Composition and Antioxidant Activity of Three Strawberry Cultivars. Food Chem..

[14] Falah M.A.F., Husna H.I., Andam Dewi A.R.P., Jumeri (2016). Quality evaluation of fresh strawberry (*Fragaria* sp. cv. Earlybrite) during storage in a tropical environment. AIP Conf. Proc..

[15] Joshi P., Pahariya P., Al-Ani M.F., Choudhary R. (2022). Monitoring and Prediction of Sensory Shelf-life in Strawberry with Ultraviolet-visible-near-infrared (UV-VIS-NIR) Spectroscopy. Appl. Food Res..

[16] Jouki M., Dadashpour A. (2012). Comparison of Physiochemical Changes in Two Popular Strawberry Cultivars Grown in Iran (Cvs. Kurdistan & Selva) during Storage Time at 4 °C. Genetika.

[17] Koyuncu M.A. (2004). Quality Changes of three Strawberry Cultivars during the Cold Storage. Europ. J. Hort. Sci..

[18] Lee C., Lee J., Lee J. (2022). Relationship of Fruit Color and Anthocyanin Content with Related Gene Expression Differ in Strawberry Cultivars during Shelf Life. Sci. Hortic..

[19] Pelayo C., Ebeler S.E., Kader A.A. (2003). Postharvest Life and Flavor Quality of Three Strawberry Cultivars Kept at 5 °C in Air or Air + 20 kPa CO_2_. Postharvest Biol. Technol..

[20] Rahman M.M., Moniruzzaman M., Ahmad M.R., Sarker B.C., Khurshid Alam M. (2016). Maturity Stages Affect the Postharvest Quality and Shelf-Life of Fruits of Strawberry Genotypes Growing in Subtropical Regions. J. Saudi Soc. Agric. Sci..

[21] Rutkowski K.P., Kruczynska D.E., Zurawicz E. (2006). Quality and shelf life of strawberry cultivars in Poland. Acta Hortic..

[22] Shin Y., Ryu J.-A., Liu R.H., Nock J.F., Watkins C.B. (2008). Harvest Maturity, Storage Temperature and Relative Humidity Affect Fruit Quality, Antioxidant Contents and Activity, and Inhibition of Cell Proliferation of Strawberry Fruit. Postharvest Biol. Technol..

[23] Feng X.Y., Wang B.G., Li W.S., Yang Y., Shi L., Yang J.J. (2014). Physical and chemical characteristics of three strawberry cultivars during cold storage. Acta Hortic..

[24] Zhang C., Guo C., Liu F., Kong W., He Y., Lou B. (2016). Hyperspectral Imaging Analysis for Ripeness Evaluation of Strawberry with Support Vector Machine. J. Food Eng..

[25] Sinanoglou V.J., Tsiaka T., Aouant K., Mouka E., Ladika G., Kritsi E., Konteles S.J., Ioannou A.-G., Zoumpoulakis P., Strati I.F. (2023). Quality Assessment of Banana Ripening Stages by Combining Analytical Methods and Image Analysis. Appl. Sci..

[26] Giannakourou M.C., Stavropoulou N., Tsironi T., Lougovois V., Kyrana V., Konteles S.J., Sinanoglou V.J. (2023). Application of Hurdle Technology for the Shelf Life Extension of European Eel (*Anguilla anguilla*) Fillets. Aquac. Fish..

[27] Nielsen S.S. (2017). Food Analysis Laboratory Manual.

[28] Andreou V., Strati I.F., Fotakis C., Liouni M., Zoumpoulakis P., Sinanoglou V.J. (2018). Herbal Distillates: A New Era of Grape Marc Distillates with Enriched Antioxidant Profile. Food Chem..

[29] Lantzouraki D.Z., Sinanoglou V.J., Zoumpoulakis P.G., Glamočlija J., Ćirić A., Soković M., Heropoulos G., Proestos C. (2015). Antiradical—Antimicrobial activity and phenolic profile of pomegranate (*Punica granatum* L.) juices from different cultivars: A comparative study. RSC Adv..

[30] Lantzouraki D.Z., Sinanoglou V.J., Zoumpoulakis P., Proestos C. (2016). Comparison of the Antioxidant and Antiradical Activity of Pomegranate (*Punica granatum* L.) by Ultrasound-Assisted and Classical Extraction. Anal. Lett..

[31] Tsiaka T., Kritsi E., Lantzouraki D.Z., Christodoulou P., Tsigrimani D., Strati I.F., Sinanoglou V.J., Zoumpoulakis P. (2022). Assessing the Phytochemical Profile and Potential of Traditional Herbal Infusions against Aldose Reductase through In Silico Studies and LC-MS/MS Analysis. Appl. Sci..

[32] Ioannou A.G., Kritsi E., Sinanoglou V.J., Cavouras D., Tsiaka T., Houhoula D., Zoumpoulakis P., Strati I.F. (2023). Highlighting the Potential of Attenuated Total Reflectance—Fourier Transform Infrared (ATR-FTIR) Spectroscopy to Characterize Honey Samples with Principal Component Analysis (PCA). Anal. Lett..

[33] Takemoto R., Koyama K., Watanabe T., Koseki S., Nakamura N. (2022). Mathematical Model for Analyzing the Effect of Storage Conditions on the Visually Perceived Freshness of Strawberries via Surface Luminance Distribution. Food Packag. Shelf Life.

[34] Muley A.B., Singhal R.S. (2020). Extension of postharvest shelf life of strawberries (*Fragaria ananassa*) using a coating of chitosan-whey protein isolate conjugate. Food Chem..

[35] Richardson A.T., Cho J., McGhie T.K., Larsen D.S., Schaffer R.J., Espley R.V., Perry N.B. (2020). Discovery of a stable vitamin C glycoside in crab apples (*Malus sylvestris*). Phytochemistry.

[36] Nyorere O. (2018). Instrumental Texture Profile Analysis (TPA) of Cucumber Fruit as Influenced by Its Part and Maturity Stage. Am. J. Eng. Technol. Manag..

[37] Nandiyanto A.B.D., Oktiani R., Ragadhita R. (2019). How to Read and Interpret FTIR Spectroscope of Organic Material. Indones. J. Sci. Technol..

[38] Anjos O., Campos M.G., Ruiz P.C., Antunes P. (2015). Application of FTIR-ATR Spectroscopy to the Quantification of Sugar in Honey. Food Chem..

[39] Kozłowicz K., Różyło R., Gładyszewska B., Matwijczuk A., Gładyszewski G., Chocyk D., Samborska K., Piekut J., Smolewska M. (2020). Identification of Sugars and Phenolic Compounds in Honey Powders with the Use of GC–MS, FTIR Spectroscopy, and X-Ray Diffraction. Sci. Rep..

[40] Minutti-López Sierra P., Gallardo-Velázquez T., Osorio-Revilla G., Meza-Márquez O.G. (2019). Chemical Composition and Antioxidant Capacity in Strawberry Cultivars (*Fragaria* × *ananassa* Duch.) by FT-MIR Spectroscopy and Chemometrics. CyTA J. Food.

[41] Brangule A., Šukele R., Bandere D. (2020). Herbal Medicine Characterization Perspectives Using Advanced FTIR Sample Techniques—Diffuse Reflectance (DRIFT) and Photoacoustic Spectroscopy (PAS). Front. Plant Sci..

[42] Bello-Pérez L.A., Ottenhof M.-A., Agama-Acevedo E., Farhat I.A. (2005). Effect of Storage Time on the Retrogradation of Banana Starch Extrudate. J. Agric. Food Chem..

[43] Wiercigroch E., Szafraniec E., Czamara K., Pacia M.Z., Majzner K., Kochan K., Kaczor A., Baranska M., Malek K. (2017). Raman and Infrared Spectroscopy of Carbohydrates: A Review. Spectrochim. Acta A Mol. Biomol. Spectrosc..

[44] Shimomura K., Horie H., Sugiyama M., Kawazu Y., Yoshioka Y. (2016). Quantitative Evaluation of Cucumber Fruit Texture and Shape Traits Reveals Extensive Diversity and Differentiation. Sci. Hortic..

[45] Oliveira R.N., Mancini M.C., Oliveira F.C.S.D., Passos T.M., Quilty B., Thiré R.M.D.S.M., McGuinness G.B. (2016). FTIR Analysis and Quantification of Phenols and Flavonoids of Five Commercially Available Plants Extracts Used in Wound Healing. Matér. Rio Jan..

[46] Canteri M.H.G., Renard C.M.G.C., Le Bourvellec C., Bureau S. (2019). ATR-FTIR Spectroscopy to Determine Cell Wall Composition: Application on a Large Diversity of Fruits and Vegetables. Carbohydr. Polym..

[47] Talari A.C.S., Martinez M.A.G., Movasaghi Z., Rehman S., Rehman I.U. (2017). Advances in Fourier Transform Infrared (FTIR) Spectroscopy of Biological Tissues. Appl. Spectrosc. Rev..

[48] Cassani L., Santos M., Gerbino E., Del Rosario Moreira M., Gómez-Zavaglia A. (2018). A Combined Approach of Infrared Spectroscopy and Multivariate Analysis for the Simultaneous Determination of Sugars and Fructans in Strawberry Juices during Storage. J. Food Sci..

[49] Grassi S., Amigo J.M., Lyndgaard C.B., Foschino R., Casiraghi E. (2014). Assessment of the Sugars and Ethanol Development in Beer Fermentation with FT-IR and Multivariate Curve Resolution Models. Food Res. Int..

[50] Mellado-Mojica E., Calvo-Gómez O., Jofre-Garfias A.E., Dávalos-González P.A., Desjardins Y., López M.G. (2022). Fructooligosaccharides as Molecular Markers of Geographic Origin, Growing Region, Genetic Background and Prebiotic Potential in Strawberries: A TLC, HPAEC-PAD and FTIR Study. Food Chem. Adv..

